# Colours’ Impact on Morality: Evidence from Event-related Potentials

**DOI:** 10.1038/srep38373

**Published:** 2016-12-22

**Authors:** Tian Gan, Wei Fang, Liezhong Ge

**Affiliations:** 1 Department of Psychology, Zhejiang Sci-Tech University, Hangzhou, 310018, China

## Abstract

Black and white have been shown to be representations of moral concepts. The purpose of this study was to investigate whether colours other than black and white have similar effects on words related to morality and to determine the time course of these effects. We presented moral and immoral words in three colours (red, green and blue) in a Moral Stroop task and used the event-related potential (ERP) technique to identify the temporal dynamics of the impact of colours on moral judgement. The behavioural results showed that it took longer for people to judge immoral words than moral words when the words were coloured green than when they were red or blue. The ERP results revealed the time course of these effects. Three stages were identified in the significant effects of P200, N300 and LPC. These findings suggest a metaphorical association between the colour green and moral information.

“Roses are red, violets are blue, sugar is sweet, and so are you.” These lines are quoted from a famous English collection of nursery rhymes. Poets use colours to express feelings and thoughts. Colours are also associated with various social and psychological meanings in everyday life. For example, red often represents competition and activity, blue implies depression and sadness, and gold represented monarchy and power in ancient China^1^,^2^.

Previous studies have revealed the effects of colour on various psychological processes, such as the ability of colours to influence emotion. Researchers have proposed that different colours are connected to different valences of emotion that have opposing effects on individuals’ behaviour^3^,^4^. Furthermore, colour can provide a clue in people’s attempts to identify the emotions depicted by facial expressions^5^. Colours also have various impacts on many cognitive processes. For example, studies have found that red can undermine people’s performance on challenging tasks that require mental manipulation and flexibility^6^,^7^,^8^. Researchers have proposed that red may evoke individuals’ avoidance because it reminds people of failure or danger^9^. In contrast, some experiments have found evidence that green and blue may be beneficial for creative processing^9^,^10^. Recently, an increasing number of studies have demonstrated that colours can affect social behaviours, such as communication^11^,^12^ and consumption experiences^13^,^14^,^15^,^16^. Thus, there is a comprehensive effect of colour on emotion and cognitive and social processing.

In this study, we focused on the effect of colours on words related to morality. Morality is a complex process that involves a broad spectrum of cognitive and emotional processes^17^,^18^,^19^. Previous studies have shown the effect of white and black colours on this complex process. In western countries, black is linked to evil, whereas white is linked to angels and paradise. This connection can be found in many films and, especially, in the Bible^20^. Some studies have shown that athletes dressed in black are more likely to be judged as having fouled^21^,^22^,^23^, and people’s different skin colours provoke racist behaviour in some parts of the world^24^. Previous studies have used the Stroop task to demonstrate the connection between morality and the colours of black and white and have found that participants’ speed of judgement was faster when words in black related to immorality rather than morality and when words in white related to morality rather than immorality, indicating the existence of black-immoral and white-moral associations^20^,^25^. These associations were found in English and in Chinese^26^. Previous findings have highlighted the relationships among morality and the colours black and white.

As a relatively abstract concept, why would morality be linked to a relatively concrete form such as colour? Metaphor is an important concept to explain this phenomenon. According to the conceptual metaphor theory^27^, the formation of metaphor relies on people’s early life experiences; it helps people understand abstract concepts through concrete experiences. The colour black and the concept of immorality are examples of this theory. Children are often afraid of darkness at night, and the stories they read connect the colour black to evil and monsters. Thus, when they see black, bad and immoral concepts are evoked. The opposite is true for the colour white.

In addition to black and white, other colours convey metaphorical information about morality. For example, when referring to traffic lights, it is universally known that a red light means stop and a green light means go. Red can be an indication of a serious warning, whereas green represents peaceful, healthy and permissible action. In moral judgements, we need to know what is right or wrong. Therefore, we predict that there are metaphorical associations between these colours and moral concepts: green may be associated with morality, and red may be associated with immorality. We expect the effects of these colours on moral judgement to differ. This concept has not previously been explored.

Despite the considerable interest in metaphor and the corresponding abundance of prior studies, the neural correlations of the effect of colours on morality have been poorly understood. Recently, neuroscientists demonstrated that there are many factors involved in moral processes (such as emotion^28^,^29^,^30^,^31^, culture^32^, and individual dispositions^33^) and identified the complex neural mechanisms underlying these factors. However, the neural mechanism responsible for the metaphorical connection between colours and moral concepts remains unknown. Our study attempts to answer this question. Event-related potentials (ERPs) enable researchers to follow the temporal course of moral processing due to the high temporal resolution of milliseconds. Previous ERP studies identified several components related to moral and colour processing. The first component is P200, a positive wave that peaks approximately 200 ms after stimulus onset. Some studies have revealed that P200 is connected to automatic attention attribution in early time windows^34^,^35^. Negative information, such as immoral stimuli, consistently elicit larger and shorter P200 compared to neutral and positive stimuli^36^,^37^,^38^,^39^. The second component is N300, a negative component present in the time window approximately 300 ms after target word presentation^34^,^40^. Bramão *et al*. suggested that this component is crucial for the integration of shape and colour information, which can be used to access the structural and semantic representations of an object in long-term memory^40^. A large body of ERP studies reported a late positive component (LPC) during a late time window from 300 to 800 ms post-stimulus. This component was related to mental resource distribution^41^ and was found to reflect controlled and elaborative processes, such as moral evaluation and reasoning^31^,^42^.

The previous literature has demonstrated the effect of the colours black and white on morality. However, other colours may also influence moral processes, although their effect remains unknown. The neural correlates of these effects are poorly understood. The aim of this study was to investigate the effects of colour on morality and to explore the way in which these effects occur. We used a variant of the Stroop task (the Moral Stroop task), which has been employed by other researchers. In this type of Stroop task, the experimenter asks the participants to judge the different types or valences of the words instead of the colour^20^,^26^ to evaluate the way colour influences processing in the judgement of moral words. We conducted the Moral Stroop task with ERPs to explore the neural correlates of the effects of colours on moral judgement. We predicted the following: the cognitive speed of moral judgement would be faster when words in green referred to morality rather than immorality; when words in red referred to immorality, the cognitive speed would be faster than when the words referred to morality; and this phenomenon would not exist for the colour blue. ERPs reveal the time course of these effects. According to previous studies of ERPs, P200 can indicate attention attribution at an early stage^34^,^37^. We expected that P200 could be helpful in distinguishing moral words from immoral words at an early processing stage. As N300 may be related to the semantic integration of visual features^40^, we expected a significant influence of colour on moral judgement during the time window of N300. Given that LPC may reflect the elaborative processing and moral reasoning in late time windows^31^,^42^, we predicted that the interaction between colours and morality would be significant for the LPC amplitude, which would reveal the specific distinctions of morality in different colours.

## Results

### Behavioural Results

We exclude the reaction time for incorrect trials as well as implausibly long or short RT (RTs that deviated ±3 standard deviations from the mean) trials from the analysis. In total, 4.88% of the trials were removed (3.04% incorrect and 1.84% excessively long/short response time). The accuracy and reaction time, not including the incorrect and extreme trials of each condition, are shown in Table 1. The two-way repeated-measure ANOVA of moral types and colours revealed a significant main effect of the moral type (*F* (1, 24) = 10.99, *p* < 0.01, *η*_*p*_^*2*^ = 0.31); responses to immoral words were longer than responses to moral words. The method also revealed the main effect of colours, which was also significant (*F* (2, 48) = 5.25, *p* < 0.01, *η*_*p*_^*2*^ = 0.18); the responses to green words were longer than responses to red and blue words. Most importantly, the interaction between moral types and colours reached significance (*F* (2, 48) = 7.33, *p* < 0.01, *η*_*p*_^*2*^ = 0.23). A post-hoc analysis showed that in conditions in which the words were green, the reaction time to immoral words was significantly longer than it was to moral words. However, this difference was not significant in conditions in which the words were red or blue. The positive and negative affect scale (PANAS) result suggested that the evaluation of emotion before and after the Moral Stroop task did not differ (see Table 2).

### ERP Results

#### Amplitude of P200

The ANOVA for amplitudes of P200 revealed significant main effects of areas (*F* (4, 96) = 35.06, *p* < 0.001, *η*_*p*_^*2*^ = 0.59), showing that the largest P200 was measured in the frontal area (12.55 ± 4.60 μV). The main effect of electrodes was significant (*F* (4, 96) = 15.28, *p* < 0.001, *η*_*p*_^*2*^ = 0.15), indicating that the middle electrodes had the largest P200 (11.85 ± 4.70 μV). The main effect of moral types was significant (*F* (1, 24) = 20.17, *p* < 0.001, *η*_*p*_^*2*^ = 0.46), with the immoral words (11.33 ± 4.64 μV) eliciting larger P200 than the moral words (10.72 ± 3.89 μV). The main effect of colours was also significant (*F* (2, 48) = 35.06, *p* = 0.190, *η*_*p*_^*2*^ = 0.15), with green words eliciting the largest P200 (11.55 ± 4.34 μV). The interaction between areas and electrodes was significant (*F* (16, 384) = 12.65, *p* < 0.001, *η*_*p*_^*2*^ = 0.35), indicating that the P200 amplitude measured at the Fz site was the largest (12.62 ± 6.48 μV). A significant interaction between areas and colours was found (*F* (8, 192) = 8.77, *p* < 0.001, *η*_*p*_^*2*^ = 0.27). A post-hoc analysis showed that over the frontal and fronto-central areas, green words (12.93 ± 4.76 μV; 12.25 ± 5.07 μV) elicited larger P200 than blue (11.76 ± 6.07 μV; 11.10 ± 6.01 μV) and red words did (12.24 ± 4.82 μV; 11.55 ± 4.92 μV). In the central area, green words (11.18 ± 5.04 μV) elicited larger P200 than blue words did (10.22 ± 5.36 μV). The interaction of areas, electrodes and moral types was also significant (*F* (16, 384) = 2.65, *p* = 0.001, *η*_*p*_^*2*^ = 0.10). The post-hoc tests revealed that immoral words elicited larger P200 than moral words in all areas, and the largest difference between immoral and moral words was measured at the Cz site.

#### Latency of P200

The ANOVA for latencies of P200 revealed a significant main effect of areas (*F* (4, 96) = 19.02, *p* < 0.001, *η*_*p*_^*2*^ = 0.44), with the shortest P200 latency measured in the frontal area (233 ± 41 ms). The main effect of electrodes was also significant (*F* (4, 96) = 5.17, *p* = 0.001, *η*_*p*_^*2*^ = 0.18), with the shortest P200 latency measured in the middle electrodes (256 ± 44 ms). The interaction between areas and electrodes was significant (*F* (16, 384) = 1.74, *p* = 0.038, *η*_*p*_^*2*^ = 0.07), indicating that the shortest P200 latency was measured at the FCz (214 ± 54 ms) site. The interaction between areas and moral types was significant (*F* (4, 96) = 3.59, *p* = 0.009, *η*_*p*_^*2*^ = 0.13). In the parietal area, the P200 latencies of immoral words were shorter than those of moral words. The three-way interaction of areas, electrodes and moral types was also significant (*F* (16, 384) = 2.00, *p* = 0.012, *η*_*p*_^*2*^ = 0.08). The LSD post-hoc tests showed that immoral words elicited shorter P200 latencies than moral words did at electrodes in the centro-parietal and parietal areas (e.g., CP2, P2, Pz).

#### Amplitude of N300

The ANOVA for amplitudes of N300 revealed a significant main effect of areas (*F* (4, 96) = 5.20, *p* = 0.001, *η*_*p*_^*2*^ = 0.18), with the largest N300 measured in the centro-parietal area (3.26 ± 3.23 ms). The interaction of areas and electrodes was also significant (*F* (16, 384) = 6.78, *p* < 0.001, *η*_*p*_^*2*^ = 0.22), indicating that the largest N300 was measured at the Fz (1.09 ± 3.89 μV) site. The main effect of colours was significant (*F* (2, 48) = 12.87, *p* < 0.001, *η*_*p*_^*2*^ = 0.35), because the N300 of the red (1.93 ± 3.42 μV) and blue (1.59 ± 3.43 μV) words was larger than that of the green words (3.14 ± 2.99 μV). There was a significant interaction between areas and colours (*F* (8, 192) = 5.84, *p* < 0.001, *η*_*p*_^*2*^ = 0.20). Blue and red words elicited larger N300 than green words did in all areas.

#### Latency of N300

The ANOVA for the latencies of N300 revealed a significant main effect of the areas (*F* (4, 96) = 11.75, *p* < 0.001, *η*_*p*_^*2*^ = 0.33), with the shortest N300 latency measured in the parietal area (295 ± 21 ms). The main effect of colours was significant (*F* (2, 48) = 3.72, *p* = 0.031, *η*_*p*_^*2*^ = 0.13). The N300 latency of the red words (299 ± 20 ms) was shorter than that of the green words (305 ± 20 ms). Most importantly, the interaction of the areas, colours and moral types was significant (*F* (8, 192) = 2.88, *p* = 0.005, *η*_*p*_^*2*^ = 0.11). The post-hoc tests showed that in the centro-parietal area, the N300 latencies of immoral words were longer than those of moral words only when the words were green.

#### Amplitude of LPC

The ANOVA for amplitudes of LPC revealed significant main effects of areas (*F* (4, 96) = 20.45, *p* < 0.001, *η*_*p*_^*2*^ = 0.30), indicating that the largest LPC was measured in the central area (10.93 ± 2.91 μV). The main effect of electrodes was significant (*F* (4, 96) = 26.91, *p* < 0.001, *η*_*p*_^*2*^ = 0.53), indicating that the largest LPC was measured in the middle electrodes (11.03 ± 3.28 μV). The interaction between areas and electrodes was significant (*F* (16, 384) = 18.20, *p* < 0.001, *η*_*p*_^*2*^ = 0.43), with the largest LPC measured at the Cz (12.28 ± 3.97 μV) site. The main effect of moral type was significant (*F* (1, 24) = 20.19, *p* < 0.001, *η*_*p*_^*2*^ = 0.47), with immoral words (11.05 ± 3.05 μV) eliciting larger LPC than moral words (9.39 ± 2.94 μV). More importantly, the interaction between moral types and colours reached significance (*F* (2, 48) = 4.26, *p* = 0.02, *η*_*p*_^2^ = 0.15). The post-hoc tests showed that the differences between immoral and moral words in green were significantly larger than the differences for words in red and blue. The interaction of moral types, colours and electrodes was also significant (*F* (8, 192) = 2.01, *p* = 0.047, *η*_*p*_^*2*^ = 0.08). The post-hoc tests showed that immoral words elicited a larger LPC than moral words did in green at all electrodes. However, at some electrodes (e.g., F1, C1, P1), the differences between immoral and moral words did not reach significance in red and blue.

#### Latency of LPC

The ANOVA for latencies of LPC revealed a significant main effect of areas (*F* (4, 96) = 13.74, *p* < 0.001, *η*_*p*_^*2*^ = 0.36), with the shortest LPC latency measured in the parietal area (494 ± 24 ms). The interaction between areas and electrodes was significant (*F* (16, 384) = 1.75, *p* = 0.037, *η*_*p*_^*2*^ = 0.07). The latencies of LPC at the parietal electrodes were the shortest (e.g., P4). The main effect of the moral types was significant (*F* (1, 24) = 5.83, *p* = 0.024, *η*_*p*_^*2*^ = 0.20), and the LPC latencies of immoral words (512 ± 29 ms) were longer than those of moral words (499 ± 27 ms). The three-way interaction of moral types, areas and electrodes was significant (*F* (16, 384) = 2.16, *p* = 0.006, *η*_*p*_^2^ = 0.08). The post-hoc tests showed that the LPC latencies of immoral words were longer than those of moral words at many electrodes in the central, centro-parietal and parietal areas (e.g., Cz, CPz, Pz, *et al*.).

**Figure 1:**
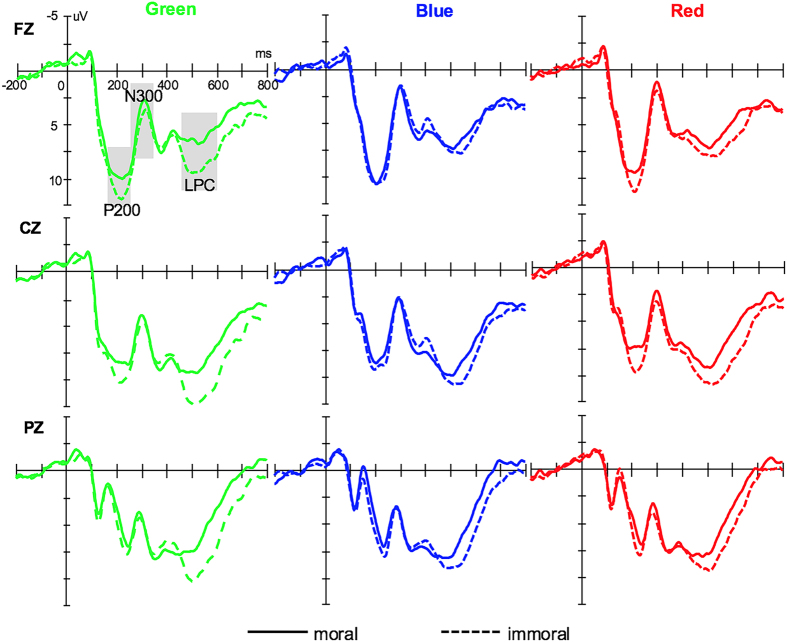
Overview of grand-averaged ERP waveforms, green-immoral and green-moral (green), red-immoral and red-moral (red), blue-immoral and blue-moral (blue) at example electrodes (Fz, Cz and Pz). The grey areas indicate time windows chosen for the analysis (P200:160–260 ms; N300:250–350 ms; LPC:450–600 ms).

**Figure 2:**
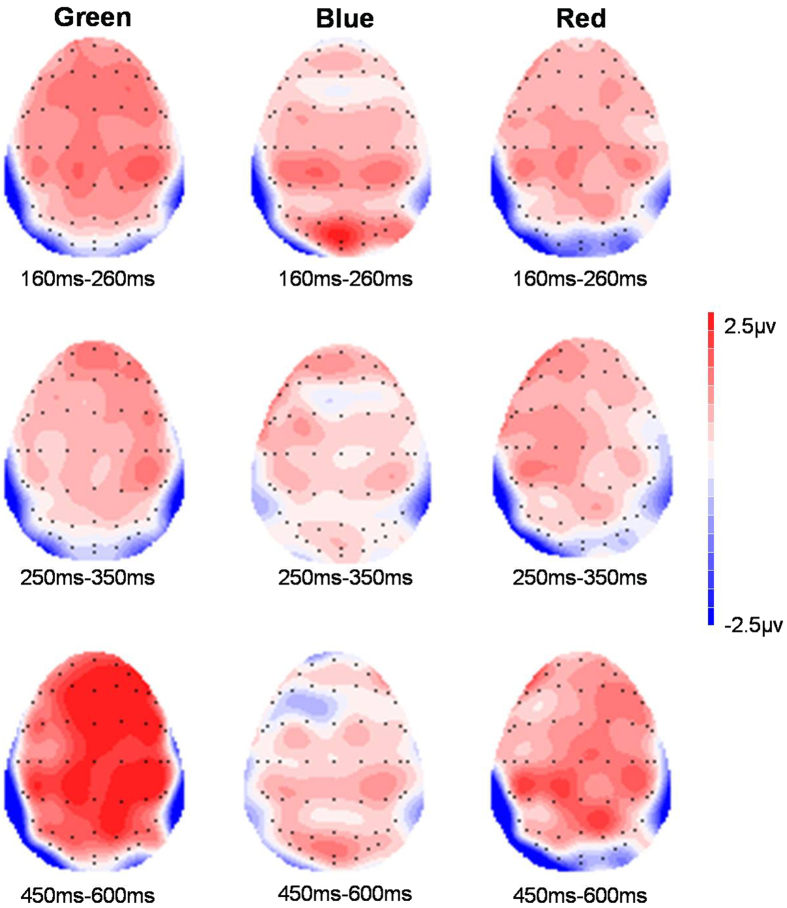
Topographical scalp distribution of the difference waves computed by subtracting the moral words from the immoral words in the time windows of 160–260 ms after the stimulus onset (top), 250–350 ms after the stimulus onset (middle) and 450–600 ms after the stimulus onset (bottom). Note that the greater positivity displayed in the time window of 450–600 ms reflecting the difference between immoral words and moral words is much higher in the green tests than in the red and blue tests.

Figure 1 shows the overview of grand-averaged ERP waveforms at example electrodes (Fz, Cz and Pz). To summarize the electrophysiological results, topographical maps displaying significant ERP differences between immoral and moral words when they were green, blue and red in the specific time windows of their ERP components are shown in Fig. 2.

## Discussion

In the present study, we conducted an ERP experiment using the Moral Stroop task to explore the impact of colours on the processing of words related to morality. According to the predictions, the behavioural results revealed a significant effect on the reaction time to moral types; immoral words were recognized at a much slower rate than moral words were. More importantly, the significant interaction between moral types and colours showed that the differences in reaction time between moral and immoral words reached significance only when the words were green instead of other colours. These results demonstrated the different effects of colours on morality, especially the impact of green on moral judgement, despite the fact that the stimulus colour was completely irrelevant to the task. According to previous studies, green has a metaphorical meaning that is often peaceful and positive^43^,^44^. These meanings were contradicted in terms of the immoral concept, which, as a result, led to a slower reaction among participants in recognizing green immoral words compared with green moral words. However, the differences in the participants’ reaction time between moral and immoral words did not reach significance when the words were red or blue. We suggest that this is because red and blue have multiple contradictory metaphorical representations. Red usually represents danger and errors. For example, warning signs are often red. However, in China, red is often associated with enthusiasm, happiness and love. One specific shade of red known as “Chinese red” is traditionally used for celebratory occasions. Similarly, we can link blue to depression and frustration. However, blue can also evoke feelings of peace and relaxation. These metaphorical representations of colours were consistent with previous findings. Zhang *et al*. conducted a Free Association Test to investigate Chinese people’s associations with various colours. Their results showed that blue and red could be associated with both positive and negative conceptions, whereas green could be associated with positive conceptions^43^. Huang *et al*. also found that Chinese people have both positive and negative associations with red^44^. These interpretations of red and blue may eliminate some subjective judgements between moral and immoral concepts.

The second aim of this study was to investigate the temporal course of the brain activation of moral judgement under the impact of different colours. From 200 ms to 1600 ms post-stimulus, three ERP components were found, and the effects of these components suggested three processing stages during moral judgement.

In the first stage, a positive component peak approximately 200 ms post-stimulus showed the main effects of moral types and colours. Consistent with our expectation, compared to moral words, immoral words elicited a larger and faster P200. These findings were consistent with previous studies of ERP on morality. For instance, Leuthold *et al*. reported a larger P200 for a word that involved a socio-normative violation^36^. Wang *et al*. found that impersonal dilemmas elicited larger P200 than personal dilemmas did in moral judgement^32^. Previous studies have shown that the P200 component is related to attention^34^,^35^, which is especially sensitive to negative visual stimulus^37^,^38^. The larger and faster P200 elicited by immoral words in this study suggested that compared to moral information, immoral information is noticed much more quickly and may attract more attention in this early stage. Another effect of P200 was that the amplitude of green words was larger than that of blue and red words. Previous studies have investigated people’s ocular sensitivity to different colours and found that human eyes are most sensitive to green and least sensitive to blue^45^,^46^. According to these findings, we suggest that the sensitivity of human eyes to the colour green makes people focus more on green words than on blue and red words, as revealed by the larger P200. The main effects of moral types and colours on P200 indicate that during this early stage, the human brain processes colours and morality information in parallel.

In the continuation of the process, in the second stage approximately 300 ms post-stimulus, the amplitudes of N300 demonstrated a significant main effect of colours, with red and blue words eliciting more negative N300 than green words. In a review of previous studies, N300 has been associated with perceptual as well as semantic processing^47^,^48^. Lu *et al*. conducted an ERP study to investigate the role of colour knowledge in the perceptual process^34^. They found that incongruently coloured pictures (e.g., a blue apple) were associated with more negative N300 than congruently coloured pictures were (e.g., red apple) and suggested that N300 may be related to the integration of perceptual information (e.g., colour and shape) with semantic processing. Similarly, Bramão *et al*. found that atypically coloured objects can elicit more negative N300 compared to typically coloured objects^40^. In this study, the colours red and blue both had two metaphorical meanings. The larger N300 under these two conditions showed the complex processing of integrating the colours’ metaphorical meanings with the words’ moral semantic information. For the latency of N300, the interaction of areas, moral types and colours was significant. Specifically, in the centro-parietal area, the N300 latencies of immoral words were longer than those of moral words only in the green condition. This effect was consistent with our expectation and suggested that it took longer to integrate the metaphorical meaning of the colour green with immoral information compared to moral information.

In the third stage, during the late time window, the LPC showed a significant main effect of moral types on amplitude and latency. Immoral words elicited larger and longer LPCs than moral words did. The differences of the LPC between moral and immoral stimuli have been demonstrated in previous studies. Van *et al*. found that value-inconsistent words elicited larger LPCs than value-consistent words did and suggested that strongly disagreeable statements automatically require additional processing resources^49^, just as negatively valenced words and pictures do^39^,^50^,^51^. Recently, Hundrieser and Stahl found a higher amplitude of LPC for value-incongruent words compared to value-congruent words in moral sentences with extreme topics and suggested that the LPC is rather sensitive to emotionally intense or arousing events^52^. Leuthold *et al*. also reported a larger LPC that was elicited by morally unacceptable words compared to acceptable words and used this effect to reflect on the implicit evaluation processing in moral judgement^36^. Based on these findings, the larger and longer LPCs of immoral words indicate that people require more time and distribute more mental resources to manage immoral stimuli compared to moral stimuli. Another important finding regarding LPCs was the interaction effect between moral types and colours. The amplitude differences between immoral and moral words were significantly larger in green than in red and blue. This ERP effect was consistent with the behavioural findings that the reaction time differences between moral and immoral words only reached significance when the words were green rather than other colours. Previous studies have suggested that LPC is related to mental resource distribution^41^ and have considered it a representation of slow but controlled and elaborative processes, such as moral evaluation and reasoning^31^,^36^,^42^. The larger LPC differences in green words in the present study showed that the conflict between immoral words and the metaphorical meanings of the colour green might lead people to devote more cognitive resources to completing moral evaluations and reasoning processes in late time windows.

In summary, this study investigated the impact of colours on words related to morality. Specifically, the behavioural results revealed the special effect of the colour green on moral judgement and demonstrated consistency between the metaphor of green and morality and inconsistency between the metaphor of green and immorality. This finding is a good complement to research on the association between morality and metaphors related to colours, which has expanded our understanding of how perception influences moral processing. Our results were consistent with the study by Lakoff and Johnson, who suggested that this concept was based on physical metaphors^53^. Colours, as one type of physical information, relate to different metaphors. When people process the conception of morality, these various metaphors provide a link between colours and morality. When the association is consistent, the reaction is much easier and faster; when it is not, the reaction is much more difficult and slower. The ERP results further revealed the time course and neural mechanism behind this association. To the best of our knowledge, this is the first study to use ERPs to assess the temporal dynamics of the impact of colour on moral judgement. Three stages may be involved in moral judgement in the Stroop task. The first stage involves early attention attribution to morality and colours. The main effects of colours and moral types on the amplitudes of P200 suggested that during this stage, the processing of basic visual features and the processing of social information may be parallel. The second stage involves the integration of perceptual information with semantic processing, which was indexed by the interaction of colours and moral type on N300. The longer N300 latency of immoral words compared to moral words in the green condition revealed the longer time interval that people require to integrate the conflicting meanings between green’s metaphorical and immoral information. This interaction lasted until the third stage, which involves elaborative evaluation and reasoning processing. The larger LPC differences in green words compared to red and blue words showed that a large amount of cognitive resources was devoted to resolving the conflict between green’s metaphorical association and immoral words during the moral evaluation and reasoning processing stage. The ERP effects revealed the impact of colours on morality, from attention, visual features and semantic processing to advanced moral evaluation and reasoning.

There are some limitations of this study. First, the present study demonstrates only the impact of colours on the moral judgement of words. Future studies should investigate whether these impacts exist in more complex situations, such as moral dilemmas or jury judgements. Second, previous studies have demonstrated the influence of colour on emotion^5^. Emotion plays an important role in moral judgement^29^. Is the impact of colour on morality related to the effect of emotion? Since we did not find significant differences in the PANAS evaluations before and after moral judgement in the present study, the possible role of emotion in the effect of colours on morality is unknown and should be explored in future studies. Finally, this study investigated only three types of colours. The impact of other colours, such as gold and yellow, remains unexplored. We manipulated only the hue as a variable in the study using brightness and saturation, which may influence moral judgement^54^,^55^,^56^. This issue will be explored in future studies.

## Materials and Methods

### Participants

After excluding 2 participants due to the lack of efficient trials, a sample of 25 participants (13 males, mean age = 20.5 years, SD = 2.5) with normal or corrected-to-normal visual acuity and no history of neurological diseases or colour blindness remained for the data analysis. All participants were right-handed, and informed written consent was obtained from each participant. All experimental methods were conducted in accordance with the approved guidelines regarding all relevant aspects, including recruitment, experimental process information, compensation and debriefing of participants. Ethical approval for the study was obtained from the Research Ethics Committee of the Zhejiang Sci-Tech University, where this experiment was conducted.

### Materials

We selected two categories of Chinese words, immoral words (e.g., evil, violence) and moral words (e.g., kind, honest). Each category consisted of 60 words. All of these words were high frequency words according to the “Frequency dictionary of the Chinese Language” (1986). Twenty participants (9 males, mean age = 22.5 years, SD = 1.3) who were not part of the ERP experiment were asked to judge the morality of these words on a scale from 1 (extremely immoral) to 7 (extremely moral) before the experiment. The independent-sample t-tests showed that the immoral words (M = 2.07, SD = 0.96) were rated at a significantly higher level (immoral) than the moral words were (M = 6.12, SD = 0.94), t = 85.45, *p* < 0.001. We also conducted independent-sample t-tests to compare the familiarity of immoral words and moral words on a scale from 1 (extremely unfamiliar) to 5 (extremely familiar). The findings showed that there was no significant difference between immoral words (M = 4.24, SD = 0.17) and moral words (M = 4.27, SD = 0.27) in terms of familiarity (20 participants, 8 males, mean age = 22.7, SD = 1.4).

The word colours in each trial were selected from a colour spectrum based on the HSB colour model^55^. The HSB allows users to choose a colour with a hue from 0° to 360° and with saturation and brightness values from 0% to 100%. All the colours we chose were fully saturated (100% saturation) and extremely bright (100% brightness). The angle for the hue was defined as the angle relative to pure red on the colour circle. The hues of the colours we chose were red (0°), green (135°), and blue (225°), based on previous studies^55^,^56^. Each word appeared three times in each of the colours (red, green and blue). The word (the horizontal visual angle was 1.56°, and the vertical visual angle was 0.78°) was located at the centre of the computer screen with a white background.

### Procedure

The experiments were conducted in a dimly lit, soundproofed room. The participants were seated on a comfortable chair with their eyes approximately 90 cm away from a 17-inch computer screen. In each trial, after a fixed cross was presented on screen randomly from 300 ms to 700 ms, the target word was presented until a response was made or for a maximum of 2000 ms. The participants were instructed to indicate whether the word was related to morality or immorality by pressing the “F” or “J” key as quickly and as accurately as possible. The key assignment was counterbalanced across the participants. The inter-trial interval was 1000 ms. The experiment included 360 trials, and the sequence of the trials was random. The participants were allowed to take a break after 120 trials. To monitor emotion, each participant was asked to complete the positive and negative affect scale (PANAS)^57^,^58^ before and after the Stroop task.

### EEG Recording and Analysis

An electroencephalogram (EEG) recorded data from 64 scalp sites using tin electrodes mounted in an elastic cap (NeuroScan Inc.) with an online reference to the right mastoid. A horizontal electrooculogram (EOG) was recorded from the electrodes placed at the outer canthi of both eyes. A vertical EOG was recorded from electrodes placed above and below the left eye. All interelectrode impedances were maintained under 5 KΩ. The EEG and EOG signals were amplified with a 0.05–100 Hz bandpass filter and were continuously sampled at 500 Hz/channel.

During the offline analysis, the EEG was re-referenced to the average of the right and left mastoids. The ocular artefacts were removed from the EEG signal using a regression procedure implemented in the Neuroscan software^59^. The EEG was averaged in 1600 ms epochs (200-ms baseline) that were time-locked to the presentation of the target word. These averages were digitally filtered with a 30 Hz low-pass filter and were baseline corrected by subtracting the average activity of that channel during the baseline period from each sample. Any trials in which the EEG voltages exceeded a threshold of ±80 μV during the recording epoch were excluded from the analysis.

Based on the hypothesis and visual inspection of the grand averaged ERPs (Fig. 1), the peak amplitudes and latencies of P200 (160–260 ms), N300 (250–350 ms) and LPC (450–600 ms) were investigated in their respective time windows. Twenty-five electrode sites over five areas were selected for a statistical analysis of these components (frontal: F3, F1, Fz, F2, F4; fronto-centro: FC3, FC1, FCz, FC2, FC4; central: C3, C1, Cz, C2, C4; centro-parietal: CP3, CP1, CPz, CP2, CP4; and parietal: P3, P1, Pz, P2, P4).

A four-way repeated measure analysis of variance (ANOVA) was conducted using the following variables as within-subject factors: moral types (two levels: moral, immoral), colours (three levels: red, green, blue), areas (five levels: frontal, fronto-central, central, centro-parietal, and parietal) and electrodes (five levels: left, left-middle, middle, right-middle, and right electrodes). The Greenhouse-Geisser correction was adopted when the spherical assumption was violated. Post-hoc testing of significant main effects and interactions was conducted using the least significant difference (LSD) method.

## Additional Information

**How to cite this article**: Gan, T. *et al*. Colours’ Impact on Morality: Evidence from Event-related Potentials. *Sci. Rep.* **6**, 38373; doi: 10.1038/srep38373 (2016).

**Publisher's note:** Springer Nature remains neutral with regard to jurisdictional claims in published maps and institutional affiliations.

## Figures and Tables

**Table 1:** Mean reaction time (RT) in ms (incorrect and extreme trials were excluded) and accuracy (ACC) to each condition, SD in parentheses.

	Moral words		Immoral words	
	RT	ACC	RT	ACC
Green	605 (72)	0.97 (0.03)	643 (85)	0.96 (0.03)
Red	611 (70)	0.97 (0.04)	617 (80)	0.98 (0.02)
Blue	608 (72)	0.98 (0.02)	621 (83)	0.96 (0.03)

**Table 2:** Means and standard deviations (SD) of assessments in the PANAS.

Type of emotion	Before		After		*t* (*25*)	*p*
	Mean	SD	Mean	SD		
Positive	2.75	0.67	2.69	0.74	0.63	0.53
Negative	1.42	0.39	1.31	0.32	1.69	0.10

## References

[b1] Aslam M. M. Are you selling the right color? A cross-cultural review of colour as a marketing cue. J Mark Commun. 12, 15–30 (2006).

[b2] Zhang T. X. & Han B. X. Psychological Effect of Red: Phenomenon and Mechanism Review. AdvPsycholSci. 21, 398–406 (2013).

[b3] Buechner V. L., Maier M. A., Lichtenfeld S. & Elliot A. J. Emotion expression and color: Their joint influence on perceived attractiveness and social position. Curr Psychol. 34, 422–433 (2015).

[b4] Ott J. W. The dual function of the eyes. South Joptom. 21, 8–13 (1979).

[b5] Silver H. & Bilker W. B. Colour influences perception of facial emotions but this effect is impaired in healthy ageing and schizophrenia. Cogn Neuropsychiatry. 20, 438–455 (2015). 26395165 10.1080/13546805.2015.1080157

[b6] Elliot A. J. . Color and psychological functioning: the effect of red on performance attainment. J Exp Psychol Gen. 136, 154–168 (2007). 17324089 10.1037/0096-3445.136.1.154

[b7] Bertrams A., Baumeister R. F., Englert C. & Furley P. Ego depletion in color priming research: self-control strength moderates the detrimental effect of red on cognitive test performance. Pers Soc Psychol B, 41, 311–322 (2015). 10.1177/0146167214564968 25567999

[b8] Meier M. A. . Color in achievement context in humans.in Handbook of color psychology, 568–584 (University University Press, 2015).

[b9] Mehta R. & Zhu R. J. Blue or red? Exploring the effect of color on cognitive task performance. Science, 323, 1226–29 (2009). 19197022 10.1126/science.1169144

[b10] Lichtenfeld S., Elliot A. J., Maier M. A. & Pekrun R. Fertile green green facilitates creative performance. Pers Soc Psychol B, 38, 784–797 (2012). 10.1177/0146167212436611 22427383

[b11] Meier B. P. . Color in context: Psychological context moderates the influence of red on approach-and avoidance-motivated behavior. PloS one. 7, e40333 (2012). 22808136 10.1371/journal.pone.0040333 PMC3394796

[b12] Guéguen N. & Jacob C. Lipstick and tipping behavior: when red lipstick enhance waitresses tips. Int J Hosp Manag. 31, 1333–1335 (2012).

[b13] Alberts W. A. & Geest T. M. V. D. Color matters: color as trustworthiness cue in web sites. Tech Commun 58, 149–160 (2011).

[b14] Gorn G. J., Chattopadhyay A., Sengupta J. & Tripathi S. Waiting for the web: how screen color affects time perception. J Marketing Res. 41, 215–225 (2004).

[b15] Lee S. & Rao V. S. Color and store choice in electronic commerce: the explanatory role of trust. J Electr Commer Res, 11, 110–126 (2012).

[b16] Yüksel A. Exterior color perceived retail crowding: effects on tourists’ shopping quality inferences and approach behaviors. J Qual Assur Hosp Tour 10, 233–254 (2009).

[b17] Haidt J. The new synthesis in moral psychology. Science 316, 998–1002 (2007). 17510357 10.1126/science.1137651

[b18] Decety J., Michalska K. J. & Kinzler K. D. The contribution of emotion and cognition to moral sensitivity: a neurodevelopmental study. Cereb Cortex 22, 209–220 (2012). 21616985 10.1093/cercor/bhr111

[b19] Feldman Hall O., Mobbs D. & Dalgleish T. Deconstructing the brain’s moral network: dissociable functionality between the temporoparietal junction and ventro-medial prefrontal cortex. Soc Cogn Affect Neur 9, 297–306 (2014). 10.1093/scan/nss139 PMC3980797 23322890

[b20] Meier B. P. & Robinson M. D. Why the sunny side is up associations between affect and vertical position. Psychol Sci. 15, 243–7 (2004). 15043641 10.1111/j.0956-7976.2004.00659.x

[b21] Frank M. G. & Gilovich T. The dark side of self-and social perception: black uniforms and aggression in professional sports. J Pers Soc Psychol 54, 74–85 (1988). 3346809 10.1037//0022-3514.54.1.74

[b22] Webster G. D., Urland G. R. & Correll J. Can uniform color color aggression? Quasi-experimental evidence from professional ice hockey. Soc Psychol Personal Sci. 3, 274–281 (2012).

[b23] Krenn B. The impact of uniform color on judging tackles in association football. Psychol Sport Exerc 15, 222–225 (2014).

[b24] Wittenbrink B., Judd C. M. & Park B. Evaluative versus conceptual judgments in automatic stereotyping and prejudice. J Exp Soc Psychol 37, 244–252 (2001).

[b25] Sherman G. D. & Clore G. L. The color of sin white and black are perceptual symbols of moral purity and pollution. Psychol Sci. 20, 1019–1025 (2009). 19619180 10.1111/j.1467-9280.2009.02403.x PMC2832478

[b26] Yin R. & Ye H. S. The Black and White Metaphor Representation of Moral Concepts and Its Influence on Moral Cognition. Act Psychol Sci. 46, 1331–1346 (2014).

[b27] Lakoff G. & Johnson M. Metaphors we live by (University of Chicago press,1980).

[b28] Christensen J. F. & Gomila A. Moral dilemmas in cognitive neuroscience of moral decision-making: a principled review. Neurosci Biobehav Rev. 36, 1249–1264 (2012). 22353427 10.1016/j.neubiorev.2012.02.008

[b29] Greene J. D. . An fMRI investigation of emotional engagement in moral judgment. Science 293, 2105–2108 (2001). 11557895 10.1126/science.1062872

[b30] Sarlo M. . Temporal dynamics of cognitive–emotional interplay in moral decision-making. J Cognitive Neurosci. 24, 1018–1029 (2012). 10.1162/jocn_a_00146 21981668

[b31] Gui D. Y., Gan T. & Liu C. Neural evidence for moral intuition and the temporal dynamics of interactions between emotional processes and moral cognition. Soc Neurosci 1–15 (2015). 10.1080/17470919.2015.1081401 26286634

[b32] Wang Y., Deng Y., Sui D. & Tang Y. Y. Neural correlates of cultural differences in moral decision making: a combined ERP and sLORETA study. Neuroreport 25, 110–116 (2014). 24366325 10.1097/WNR.0000000000000077

[b33] Yoder K. J. & Decety J. Spatiotemporal neural dynamics of moral judgment: A high-density ERP study. Neuropsychologia 60, 39–45 (2014). 24905282 10.1016/j.neuropsychologia.2014.05.022 PMC4104265

[b34] Lu A. . Electrophysiological evidence for effects of color knowledge in object recognition. Neurosci Lett. 469, 405–410 (2010). 20026380 10.1016/j.neulet.2009.12.039

[b35] Missonnier P. . Working memory load–related electroencephalographic parameters can differentiate progressive from stable mild cognitive impairment. Neuroscience 150, 346–356 (2007). 17996378 10.1016/j.neuroscience.2007.09.009

[b36] Leuthold H., Kunkel A., Mackenzie I. G. & Filik R. Online processing of moral transgressions: ERP evidence for spontaneous evaluation. Soc Cogn Affect Neur 10, 1021–1029 (2015). 10.1093/scan/nsu151 PMC4526472 25556210

[b37] Carretié L. . Emotion and attention interaction studied through event-related potentials. J Cognitive Neurosci 13, 1109–1128 (2001a). 10.1162/089892901753294400 11784449

[b38] Carretié L., Mercado F., Tapia M. & Hinojosa J. A. Emotion, attention, and the ‘negativity bias’, studied through event-related potentials. Int J Psychophysiol 41, 75–85 (2001b). 11239699 10.1016/s0167-8760(00)00195-1

[b39] Huang Y. X. & Luo Y. J. Temporal course of emotional negativity bias: an ERP study. Neurosci Lett. 398, 91–96 (2006). 16446031 10.1016/j.neulet.2005.12.074

[b40] Bramão I. . The interaction between surface color and color knowledge: behavioral and electrophysiological evidence. Brain Cognition 78, 28–37 (2012). 22070924 10.1016/j.bandc.2011.10.004

[b41] Olofsson J. K., Nordin S., Sequeira H. & Polich J. Affective picture processing: an integrative review of ERP findings. Biol Psychol 77, 247–265 (2008). 18164800 10.1016/j.biopsycho.2007.11.006 PMC2443061

[b42] Chen P., Qiu J., Li H. & Zhang Q. Spatiotemporal cortical activation underlying dilemma decision-making: An event-related potential study. Biol Psychol 82, 111–115 (2009). 19576947 10.1016/j.biopsycho.2009.06.007

[b43] Zhang J. J., Liang W. T. & Huang Q. Q. On the color word association among college students. Applied Linguistics 2, 52–60 (2006).

[b44] Huang X. S. . On the color word association of Japanese college students — compared with Chinese students. Psychological Exploration 33, 146–150 (2013).

[b45] Nathans J., Thomas D. & Hogness D. S. Molecular genetics of human color vision: the genes encoding blue, green, and red pigments. Science 232, 193–202 (1986). 2937147 10.1126/science.2937147

[b46] Davis J., Hsieh Y. H. & Lee H. C. Humans perceive flicker artifacts at 500 [emsp14] Hz. Sci Rep. 5 (2015). 10.1038/srep07861 PMC4314649 25644611

[b47] McPherson W. B. & Holcomb P. J. An electrophysiological investigation of semantic priming with pictures of real objects. Psychophysiology 36, 53–65 (1999). 10098380 10.1017/s0048577299971196

[b48] Redmann A., FitzPatrick I., Hellwig F. & Indefrey P. The use of conceptual components in language production: an ERP study. Front Psychol 5, 363–363 (2014). 24808878 10.3389/fpsyg.2014.00363 PMC4010786

[b49] Van Berkum J. J. . Right or wrong? The brain’s fast response to morally objectionable statements. Psychol Sci. 20, 1092–1099 (2009). 19656340 10.1111/j.1467-9280.2009.02411.x

[b50] Cacioppo J. T. . Bioelectrical echoes from evaluative categorizations: I. A late positive brain potential that varies as a function of trait negativity and extremity. JpersSocPsychol 67, 115–125 (1994). 10.1037//0022-3514.67.1.115 8046583

[b51] Hajcak G., MacNamara A. & Olvet D. M. Event-related potentials, emotion, and emotion regulation: an integrative review. Dev Neuropsychol 35, 129–155 (2010). 20390599 10.1080/87565640903526504

[b52] Hundrieser M. & Stahl J. How attitude strength and information influence moral decision making: Evidence from event‐related potentials. Psychophysiology 53, 678–688 (2016). 26818492 10.1111/psyp.12599

[b53] Lakoff G. & Johnson M. Philosophy in the flesh: The embodied mind and its challenges to western thought 46–48 (New York: Basic Books,1999).

[b54] Chiou Wen-Bin & Cheng Ying-Yao. In broad daylight, we trust in God! Brightness, the salience of morality, and ethical behavior. J Environ Psychol 36, 37–42 (2013).

[b55] Nilgün C., Cengiz Y. & Dilek G. Effects of hue, saturation, and brightness on preference. Color ResAppl. 27, 199–207 (2002).

[b56] Nilgün C., Cengiz Y. & Dilek G. Effects of hue, saturation, and brightness: Part 2: Attention. Color Res Appl. 29, 20–28 (2004).

[b57] Watson D., Clark L. A. & Tellegen A. Development and validation of brief measures of positive and negative affect: the PANAS scales. J Pers Soc Psychol 54, 1063–1070 (1988). 3397865 10.1037//0022-3514.54.6.1063

[b58] Huang L., Yang T. Z. & Ji Z. M. Applicability of the Positive and Negative Affect Scale in Chinese. Chinese Mental Health Journal 17, 54–56 (2003).

[b59] Semlitsch H. V., Anderer P., Schuster P. & Presslich O. A solution for reliable and valid reduction of ocular artifacts, applied to the P300 ERP. Psychophysiology 23, 695–703 (1986). 3823345 10.1111/j.1469-8986.1986.tb00696.x

